# Use of Prognostic Factors and Scores in Selection of Patients with Colorectal Cancer Peritoneal Metastasis (CRPM) for Cytoreductive Surgery and Intraperitoneal Chemotherapy (CRS/IPC): Results of an International Survey Among Oncologic Clinicians

**DOI:** 10.1245/s10434-022-12794-5

**Published:** 2023-04-05

**Authors:** Mathew A. Kozman, Oliver M. Fisher, Winston Liauw, David L. Morris

**Affiliations:** 1 Hepatobiliary and Surgical Oncology Unit, Department of Surgery, St George Hospital, Kogarah, NSW Australia; 2 Cancer Care Centre, St George Hospital, Kogarah, NSW Australia; 3 St George Hospital Clinical School, University of New South Wales, Sydney, NSW Australia

## Abstract

**Background:**

No universally accepted guidelines exist for treatment of patients with colorectal cancer peritoneal metastases (CRPM) undergoing cytoreductive surgery and intraperitoneal chemotherapy (CRS/IPC). Several uncertainties remain concerning almost every aspect of this treatment modality, resulting in marked variability in patient management and likely outcomes. This survey aimed to define variations and trends in clinician decision making more clearly.

**Methods:**

A 41-question web-based survey was distributed electronically via the Peritoneal Surface Oncology Group International (PSOGI), the International Society for the Study of Pleura and Peritoneum (ISSPP) as well as via social media (particularly Twitter). The survey sought to address and record clinician responses regarding patient workup/assessment, selection for preoperative systemic therapy, preoperative and intraoperative selection for CRS/IPC, and consideration of prognosis and complications.

**Results:**

Complete responses were received from 60 clinicians from 45 centres in 22 countries. Upon assessment of survey responses, several interesting trends were noted in each section of the survey. Significant variability in surgeon practice and opinion were identified concerning almost every aspect of the treatment modality.

**Conclusion:**

This international survey provides the most comprehensive insight into clinician decision-making trends regarding patient assessment, selection and management. This should allow areas of variability to be more clearly defined and could potentially prompt development of initiatives for achieving consensus and standardisation of care in the future.

**Supplementary Information:**

The online version contains supplementary material available at 10.1245/s10434-022-12794-5.

Colorectal cancer (CRC) is the third most common cancer worldwide and the second leading cause of cancer death.^1^ The peritoneum is the third most common site of colorectal cancer metastatic spread after liver and lung, and confers the worst survival among patients with metastatic disease.^2,3^ Over the last few decades, treatment of patients with colorectal cancer with peritoneal metastases (CRPM) has evolved, with a growing body of evidence supporting cytoreductive surgery and intraperitoneal chemotherapy (CRS/IPC) as a valid treatment option which results in dramatically improved outcomes for some patient groups.^4–7^ Nonetheless, challenges exist regarding appropriate patient selection for this radical treatment, given its resource intensive nature, high frequency of tumour recurrence and potential morbidity.^4^ An international survey indicates that CRS/IPC is now performed on a large scale for patients with CRPM; however, significant variability in surgeon practice and opinion is noted on several key issues, largely due to this treatment modality not being featured in national treatment guidelines in about half the countries in which it is performed.^8^ Some authors have used a comprehensive review of the literature to create clinical algorithms to address specific patient presentations and incorporate best evidence into treatment sequencing.^9^ Nonetheless, several uncertainties remain concerning almost every aspect of this treatment modality, and no universally accepted guidelines exist, thus resulting in marked variability in patient management and likely outcomes.^10^ This emphasises the importance of defining areas of marked variability in patient selection and management in order to develop initiatives to achieve consensus and standardisation of care. Therefore, the aim of this survey was to define trends in clinician decision making regarding preoperative assessment and management, intraoperative decisions, consideration of prognosis and complications, and use of prognostic scores in patient selection.

## Materials and Methods

### Survey/Questionnaire

An extensive literature review was conducted to identify factors which potentially influence treatment choices for patients with CRPM being considered for CRS/IPC. The survey consisted of 41 questions that sought to address and record: (1) general information and level of expertise of the participant, (2) preoperative workup and assessment, (3) selection for and administration of neoadjuvant/preoperative systemic therapy, (4) factors influencing preoperative selection for CRS/IPC, (5) factors influencing intraoperative decisions to proceed with CRS/IPC and (6) consideration of prognosis and complications. The survey was reviewed by 2 surgeons and a medical oncologist with experience in CRS/IPC. A web-based survey programme and hosting site was used for developing, distributing and collecting results, as well as providing initial analysis of results (www.surveymonkey.com). Supplementary document 1 displays the survey questions. The survey design only allowed progression to the next question once a question was completed, thus only complete surveys were considered in our analysis. Participation in the survey was voluntary and all information and responses acquired were kept confidential and reported only in aggregated form.

### Distribution

The survey was distributed electronically via the Peritoneal Surface Oncology Group International (PSOGI), the International Society for the Study of Pleura and Peritoneum (ISSPP) as well as via social media (particularly Twitter). After initial distribution, a reminder was sent approximately 4–6 weeks later. The survey was open for 3 months starting on the 15th September 2021. Once this period had lapsed, the survey was closed and the database locked and collated for analysis.

### Ethics

The current study did not require ethical approval as it only includes the opinions of deidentified experts and no patient data are represented, either directly or indirectly. The study was performed in accordance with the precepts established by the Declaration of Helsinki.

### Statistical Analysis

Data were collected and analysed using Microsoft Excel. Stacked bar graphs were created where necessary to demonstrate the percentage of respondents choosing each answer. A subgroup analysis was also conducted to compare responses of those belonging to high-volume centres (≥ 50 cases per year) with those of the general cohort.

## Results

The typical time spent by each participant was 19 minutes and 30 seconds. Most scores were collated based on classical Likert scales and reported accordingly.

### Demographics and Surgical Unit Information (Questions 1–10)

As outlines in Table 1, sixty complete responses were acquired within the 3-month period. Responses were received from 22 countries: France (22, 36.7%), Australia (7, 11.7%), Sweden (6, 10.0%), New Zealand (5, 8.3%), Spain, Paraguay, Denmark (2 each, 3.3%), and Brazil, Bulgaria, Greece, India, Ireland, Israel, Italy, Japan, Romania, Saudi Arabia, Switzerland, Turkey, the United Kingdom and the United States of America (1 each, 1.7%). Responses were received from over 45 different hospitals/centres.

**Table 1:** Survey participant individual and surgical unit characteristics

	All patients (*n* = 60)
Country, *n* (%)	
France	22 (36.7)
Australia	7 (11.7)
Sweden	6 (10.0)
New Zealand	5 (8.3)
Spain	2 (3.3)
Paraguay	2 (3.3)
Denmark	2 (3.3)
Other	14 (23.8)
Gender, *n* (%)	
Male	47 (78.3)
Female	13 (21.7)
Speciality, *n* (%)	
Surgeon	59 (98.33)
Medical oncologist	1 (1.67)
Qualification, *n* (%)	
Consultant	55 (91.67)
Fellow (post fellowship training)	1 (1.67)
Trainee (resident/ registrar)	2 (3.33)
Other (e.g. researcher)	2 (3.33)
Experience (years of practice), *n* (%)	
>10	31 (56.36)
5–10	17 (30.91)
<5	7 (12.73)
Experience in CRS/IPC (years), *n* (%)	
>10	29 (48.33)
5–10	20 (33.33)
<5	11 (18.33)
Unit experience in CRS/IPC (cases/ year), *n* (%)	
>50	27 (45.00)
20–49	20 (33.33)
10–19	8 (13.33)
0–9	5 (8.33)
Use of MDT meeting, *n* (%)	
Dedicated CRS/IPC	31 (54.39)
Colorectal (lower GI)	10 (17.54)
Combined lower and upper GI	14 (24.56)
Other	2 (3.51)

Of respondents, 47 were male (78.3%) and 13 were female (21.7%). All respondents were surgeons (98.3%) apart from one medical oncologist. A vast majority of respondents were practicing consultants (91.7%) with 31 (56.4%) being in practice for over 10 years and 17 (30.9%) for 5–10 years. Twenty-nine (48.3%) of respondents had been involved in CRS/IPC for > 10 years, and 20 (33.3%) for 5–10 years. Forty-seven (78.3%) were part of a unit that performed ≥ 20 CRS/IPC operations per year. Most respondents (95.0%) belonged to a unit with a multidisciplinary team (MDT) meeting, with this being a dedicated CRS/IPC MDT for 32 (56.1%) respondents, and a gastrointestinal cancer MDT (either lower GI or combined upper and lower GI) for 24 (42.1%) respondents.

### Preoperative Assessment and Management (Questions 11–19)

For staging, all units routinely performed a CT chest/abdomen/pelvis for staging of patients with CRPM. MRI abdomen/peritoneum was used by 22 (36.7%), 40 (66.7%) used PET/CT scan, and 22 (36.7%) used MRI liver. Regarding performance of diagnostic laparoscopy, 28 (46.7%) reported always, 31 (51.7%) reported sometimes and 1 (1.7%) respondent reported never employing this staging modality. The common indications for diagnostic laparoscopy were diagnosis confirmation (biopsy), to clarify peritoneal cancer index (PCI) if uncertain on imaging and to assess resectability, while some reported routinely performing diagnostic laparoscopy if radiological PCI was 10–20.

Tumour markers were routinely used in preoperative work-up with 59 (98.3%) using CEA, 48 (80.0%) using CA19.9, 30 (50.0%) included CA125, particularly if gynaecological malignancy was suspected, 4 (6.7%) also included CA15.3. One respondent (1.7%) reported not routinely measuring any tumour markers.

Regarding consideration of systemic therapy (including neoadjuvant), 51 (85.0%) respondents reported considering use, with 24 (47.1%) administering to all patients, 16 (31.4%) to those with unresectable disease and 25 (49.0%) to those with high PCI (considered to be > 15 in the majority of respondents). Regarding administration of systemic treatment to those with metachronous vs. synchronous disease, 49 (96.1%) and 48 (94.1%) reported always/usually/sometimes administering this to patients with the respective disease pattern. Most respondents (98.0%) reported that their unit tends to prefer standard chemotherapy regimens (FOLFOX, CAPOX/XELOX/FOLFIRI/FOLFOXFIRI), 58.8% also reported use of targeted therapy, while 13.7% reported use of pressurized intraperitoneal aerosol chemotherapy (PIPAC). Fifty (98.0%) respondents routinely restaged patients post systemic therapy with similar use of imaging modalities as previously described.

### Factors Influencing Preoperative Selection for CRS/IPC (Questions 20–29)

**Fig. 1:**
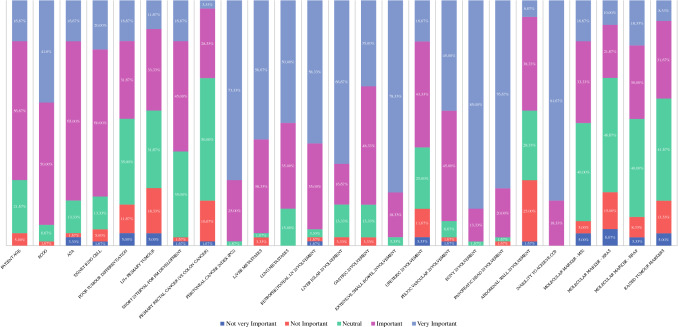
Importance of variables influencing decision to offer CRS/IPC in patients with CRPM.

When asked to consider variables influencing the decision to offer CRS/IPC to patients with CRPM, trends were noted (Fig. 1). A trend in responses toward “neutral-important” was reported for poor tumour differentiation, primary tumour lymph node status, short time from resection of primary to development of CRPM, primary rectal cancer (vs. colon cancer), abdominal wall involvement, molecular marker status (MSI/KRAS/BRAF) and raised tumour markers (CEA, CA19.9, CA125). A trend toward “important” was reported for patient age, ASA, signet-cell histology, tumour and ureteric involvement. A trend toward “important-very important” was reported for ECOG, presence of liver metastasis, presence of lung metastasis, retroperitoneal lymph node involvement, gastric involvement and pelvic vascular involvement. A trend toward “very important” was reported for PCI, liver hilar involvement, extensive small bowel involvement, bony involvement, pancreatic involvement and inability to achieve complete cytoreduction.

**Fig. 2:**
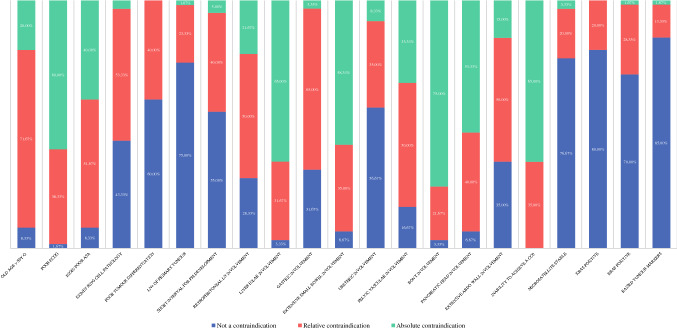
Contraindications for CRS/IPC in patients with CRPM

When asked to consider contraindications for CRS/IPC in patients with CRPM, certain trends were noted (Fig. 2). A trend in responses toward “not a contraindication” was reported for lymph node positive primary tumour, molecular markers (MS stable/KRAS positive/BRAF positive) and raised tumour markers (CEA, CA19.9, CA125). A trend toward “not a contraindication-relative contraindication” was reported for signet-cell pathology, poor tumour differentiation, short time from resection of primary tumour to development of CRPM, gastric involvement, ureteric involvement requiring reconstruction and extensive abdominal wall involvement requiring major reconstruction. A trend toward “relative contraindication” was reported for old age (80 years old) and retroperitoneal lymph node involvement. A trend toward “relative-absolute contraindication” was reported for poor ECOG status, poor ASA status, extensive small bowel involvement requiring extensive resection, pelvic vascular involvement requiring reconstruction and pancreatic head involvement. A trend toward “absolute contraindication” was reported for liver hilar involvement requiring reconstruction, bony involvement requiring resection/fixation and inability to achieve complete cytoreduction.

**Fig. 3:**
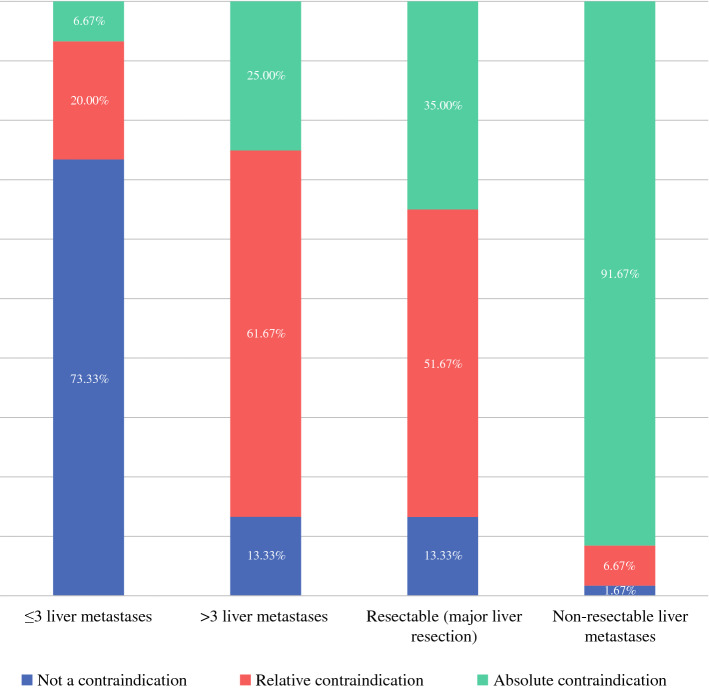
Presence of liver metastases and contraindications for CRS/IPC in patients with CRPM

**Fig. 4:**
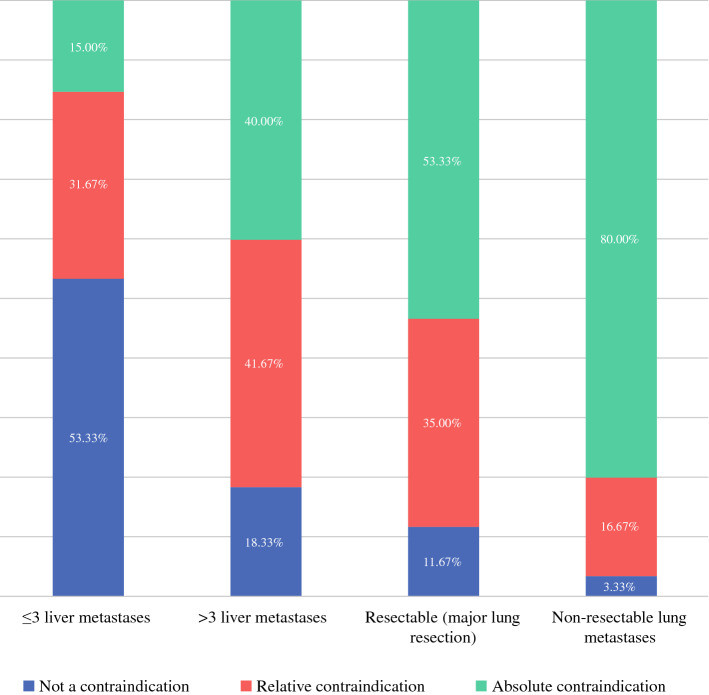
Presence of lung metastases and contraindications for CRS/IPC in patients with CRPM

Regarding PCI, 52 (86.7%) reported having a “cut-off” for offering patients treatment with CRS/IPC. Eighteen (34.6%) of those reported that PCI cuff-off was not dependent on presence of distant metastases (liver or lung), and of these the reported PCI cut-offs were ≤ 15 in 38.9%, ≤ 20 in 50% and ≤ 25 in 11.1%. The remaining thirty-four (65.4%) respondents reported that PCI cut-off was dependent on the presence of distant metastases (liver or lung). For these, the PCI cut-offs in the absence of distant metastases were ≤ 10 in 5.7%, ≤ 15 in 37.1%, ≤ 20 in 51.4% and ≤ 25 in 5.7%, and were dependent on the number of metastases. In 4 (11.8%) respondents, the presence of any metastatic disease was an absolute contraindication to CRS/IPC. Others considered < 3 liver/lung metastases to be acceptable if PCI was < 10 (19, 55.9%), < 12 (7, 20.6%) and < 15 (4, 11.8%). Many of these respondents stated that the lung/liver metastases had to be resectable/treatable to warrant proceeding with CRS/IPC. Furthermore, when asked to consider contraindications for CRS/IPC regarding the presence of liver metastases (Fig. 3), a trend in responses toward “not a contraindication” for ≤ 3 liver metastases, toward “relative contraindication” for > 3 liver metastases, toward “relative-absolute contraindication” for resectable liver metastases requiring major liver resection and toward “absolute contraindication” for non-resectable liver metastases was reported. Similar trends were reported when asked to consider contraindications for CRS/IPC regarding the presence of pulmonary metastases (Fig. 4).

Interestingly, other factors stated to influence preoperative decision making included young age, patient psychological status, patient preference and expectation, response to systemic therapy, response to PIPAC, findings on diagnostic laparoscopy, available insurance cover, other previous non-colorectal cancer, nutritional status and presence of symptoms,

### Factors Influencing Intraoperative Decisions to Proceed with CRS/IPC (Questions 30–32)

**Fig. 5:**
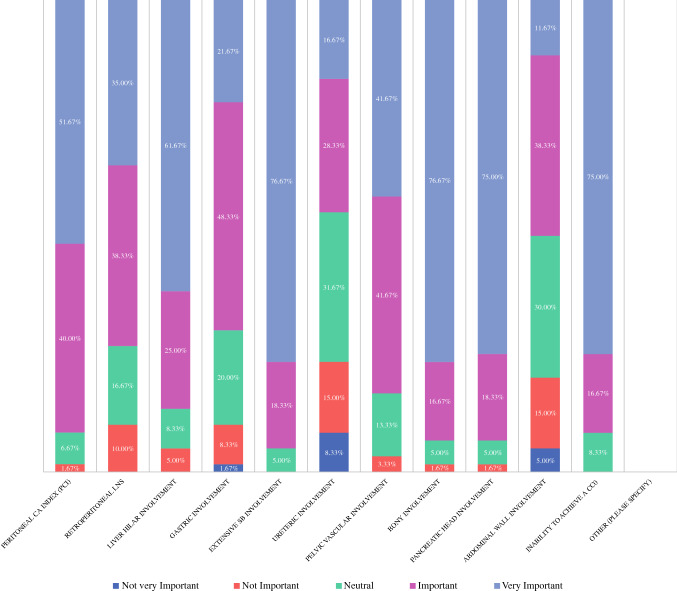
Importance of variables influencing decision to proceed intraoperatively with CRS/IPC in patients with CRPM

When asked to consider variables when deciding whether to proceed intraoperatively with CRS/IPC in patients with CRPM, certain trends were noted (Fig. 5). A trend in responses toward “neutral-important” was reported for ureteric involvement and abdominal wall involvement. A trend toward “important” was reported for gastric involvement. A trend toward “important-very important” was reported for PCI, retroperitoneal lymph node involvement and pelvic vascular involvement. A trend toward “very important” was reported for liver hilar involvement, extensive small bowel involvement, bony involvement, pancreatic head involvement and inability to achieve complete cytoreduction.

**Fig. 6:**
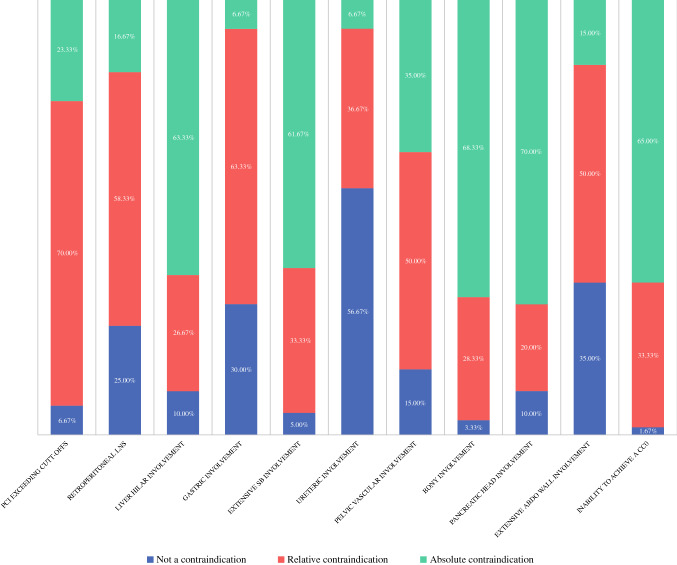
Contraindications for proceeding intraoperatively with CRS/IPC in patients with CRPM

When asked to consider contraindications for proceeding intraoperatively with CRS/IPC in patients with CRPM, certain trends were noted (Fig. 6). A trend in responses toward “not a contraindication” was reported for ureteric involvement requiring reconstruction. A trend toward “not a contraindication-relative contraindication” was reported for gastric involvement requiring resection and extensive abdominal wall involvement requiring major reconstruction. A trend toward “relative contraindication” was reported for PCI exceeding a cut-off and retroperitoneal lymph node involvement. A trend toward “relative-absolute contraindication” was reported for extensive small bowel involvement requiring extensive resection and pelvic vascular involvement requiring reconstruction. A trend toward “absolute contraindication” was reported for liver hilar involvement requiring reconstruction, bony involvement requiring resection/fixation, pancreatic head involvement and inability to achieve complete cytoreduction.

Interestingly, other factors stated to influence intraoperative decision making included patient age and comorbidities, patients’ expectations, expected functional outcomes, intraoperative complications/haemodynamic instability, presence of unexpected visceral metastases and diaphragmatic involvement requiring mesh reconstruction.

### Consideration of Prognosis and Complications (Questions 33–35)

Respondents were asked what the minimal acceptable time period (in months) would be for CRS/IPC to be warranted if prognosis could be predicted for an individual. Regarding recurrence-free survival, 31.7% said < 12 months, 45% said 12 months and 23.3% said > 12 months. Regarding overall survival, 28.3% said < 24 months, 20% said 24 months and 51.7% said > 24 months.

Respondents were asked at what percentage risk of morbidity/mortality would you recommend against CRS/IPC. Regarding Clavien-Dindo grade 3 (requiring invasive intervention) or grade 4 (requiring ICU management), the mean from respondents was 36% with 23 responding ≤ 20%. Regarding grade 5 complications (peri-operative mortality) the mean from respondents was 14% with 40 responding ≤ 5%.

Other factors which would prompt some respondents to recommend against CRS/IPC included poor mental health, poor quality of life pre-treatment, expected poor quality of life due to surgical implications, poor functional status, cognitive impairment, lack of patient motivation, chemotherapy resistance and absence of appropriate resources (interventional radiology, ICU beds). Thirty-six (60%) respondents listed no other factors.

### Use of Prognostic Scores (Questions 36–40)

Only 12 (20%) respondents reported using currently available prognostic scores (other than PCI alone) to aid with decisions regarding recommendations for CRS/IPC. Of those, 58% used the Prognostic Score (PS), 83% used the Peritoneal Surface Disease Severity Score (PSDSS), 25% used the Colorectal Peritoneal Metastases Prognostic Surgical Score (COMPASS) and 42% used the BIOlogical Score of Colorectal Peritoneal metastasis (BIOSCOPE).^11–14^ No units reported using the COloREctal-Pc (COREP) or its modification (mCOREP).^15,16^

For the remaining 48 (80%) respondents who reported not using currently available decision support tools, the reasons provided included poor accuracy (27%), tedious to calculate (35%), inability to calculate preoperatively (27%), lack of clinical relevance (33%) and lack of appropriate validation (42%).

Despite this, respondents reported that they would like to see prognostic scores/decision support tools used for prediction/prevention of open-close laparotomy (40%), identification of patients who will achieve a survival benefit from CRP/IPC (87%), prediction of response to chemotherapy (32%) and avoidance of surgery unlikely to provide a survival benefit (i.e. futile surgery) (63%).

Some relevant factors that respondents stated should be included in a prognostic score that are not already included in one of the currently available scores included frailty score, response to neoadjuvant chemotherapy (chemotherapy sensitivity), nutritional status, previous surgery, extraperitoneal metastasis status, quality of life scoring, number/site of organs involved with the disease and presence of critical lesions (porta hepatis, small bowel etc.). Several respondents emphasised the desire to see biological factors (BRAF, KRAS, MSI) included in decision-making tools developed in the future.

### Analysis of Responses from Those Belonging to High-Volume Centres

In addition to the above responses, a subgroup analysis was also conducted to compare responses of those belonging to high-volume centres (≥ 50 cases per year) with those of the entire cohort. Relevant differences are outlined in this Section.

Regarding the demographics and surgical unit information, 27 participants (45%) were from high-volume units. These were from 14 countries and 22 different hospitals/centres. It was noted that the proportion of males was lower (66.7% vs. 78.3%). The proportion of those who had been consultants for 5–10 years was considerably higher (51.9% vs. 30.9%), with a subsequent lesser proportion of those who had been consultants for over 10 years (29.6% vs. 56.4%).

Regarding preoperative workup/assessment and management, the percentage of participants who utilised MRI modalities routinely for staging was higher (MRI abdomen/peritoneum, 48.2% vs. 36.7%; and MRI liver, 44.4% vs. 36.7%). There was a reduction in the percentage of respondents who “always” used diagnostic laparoscopy for preoperative PCI calculation in this group (37.0% vs. 46.7%), with a subsequent increase in the percentage who “sometimes” used this modality for staging (63.0% vs. 51.7%). The common indications for diagnostic laparoscopy were the same as those described by the general cohort. The proportion of respondents who routinely measured preoperative CEA was similar (96.3% vs. 98.3%) while those who routinely measured CA19.9 and CA125 was significantly higher (92.6% vs. 80.0%, and 63.0% vs. 50.0%, respectively).

When considering use of systemic therapy (including neoadjuvant), the percentage of respondents who considered its use (for either metachronous or synchronous disease) and indications for doing so (all patients vs. those with unresectable disease vs. those with high PCI) was similar to that seen in the general cohort, as were the preferred regimens. All respondents routinely restaged patients post systemic therapy with similar use of imaging modalities to that described by the general cohort.

Regarding factors influencing preoperative selection for CRS/IPC, more importance was placed on the following factors with percentages of those responding “very important” noted here: ASA physical status (33.3% vs. 16.7%), signet-cell pathology (29.6% vs. 20.0%) and inability to achieve CC0 cytoreduction (96.3% vs. 81.7%). In addition, more importance was placed on all molecular markers (MSI, KRAS and BRAF) and tumour markers (CEA, CA19.9, CA125) but to a lesser magnitude (i.e. < 10% difference from general cohort responses). However, less importance was placed on the following factors with percentages of those responding “very important” again noted here: PCI (63.0% vs. 73.3%), presence of liver metastases (33.3% vs. 50.0%) and extensive small bowel involvement (51.9% vs. 78.3%).

When considering contraindications for selecting patients preoperatively for CRS/IPC, the proportion of respondents that answered “absolute contraindication” was lower for every factor listed when compared with the general cohort. The magnitude of difference was highest for old age (7.4% vs. 20.0%), retroperitoneal lymph node involvement (3.7% vs. 21.7%), liver hilar involvement (51.9% vs. 65%), extensive small bowel involvement (40.7% vs. 58.3%), pelvic vascular involvement (44.4% vs. 53.3%) and extensive abdominal wall involvement (7.4% vs. 15.0%).

The percentage of respondents who reported having a PCI cut-off for offering CRS/IPC was lower than in the general cohort (74.1% vs. 85.0%), although a much higher percentage (90.0% vs. 65.4%) altered the PCI cut-off in the presence of distant metastases (liver or lung). Nonetheless, the actual PCI cut-offs reported by those respondents in both scenarios (i.e. with or without distant metastases) matched that of the general cohort. Furthermore, when asked to consider contraindications for CRS/IPC regarding the presence of liver/lung metastases, respondents were less likely to consider these to be an “absolute contraindication” across all scenarios, especially if resectable (liver, 25.9% vs. 53.3%; lung, 7.4% vs. 35.0%).

Regarding factors influencing an intraoperative decision to proceed with CRS/IPC, more importance was placed on the following factors with percentages of those responding “very important” noted here: extensive small bowel involvement (85.2% vs. 76.7%) and liver hilar involvement (66.7% vs. 61.7%). However, less importance was placed on the following factors with percentages of those responding “very important” again noted here: retroperitoneal lymph node involvement (29.6% vs. 35.0%) and ureteric involvement (7.41% vs. 16.7%).

When considering contraindications to proceed intraoperatively with CRS/IPC, the proportion of respondents that answered “absolute contraindication” was again lower for every factor listed when compared with the general cohort. The magnitude of difference was highest for PCI exceeding previously stated cut-offs (14.8 vs. 23.3%), retroperitoneal lymph node involvement (3.7% vs. 16.7%), pelvic vascular involvement (22.2% vs. 35.0%), pancreatic head involvement (59.3% vs. 70.0%) and extensive abdominal wall involvement (7.4% vs. 15.0%).

Regarding consideration of prognosis and complications, stated minimum accepted recurrence-free survival was shorter than that stated by the general cohort (< 12 months, 40.7% vs. 31.7%; ≥ 12 months, 59.3% vs. 68.3%), while for overall survival, the opposite was seen (< 24 months, 22.2% vs. 28.3%; ≥ 24 months, 77.8%% vs. 71.7%). Furthermore, acceptable percentage risk for morbidity/mortality (for Clavien-Dindo grade 3/4 and grade 5) was very similar to that stated by the general cohort.

Regarding use of prognostic scores, only 5 respondents (18.5%) reported using currently available prognostic scores (other than PCI alone) to aid with decisions regarding CRS/IPC. All used Peritoneal Surface Disease Severity Score (PSDSS) with 3 (60.0%) also using PS and 2 (40%) using BIOSCOPE. For those who reported not using these decision support tools, the reasons offered were the same as for those of the general cohort. Lastly, stated desired purposes for the future utility of prognostic scores were also the same as those of the general cohort.

## Discussion

To the best of our knowledge, this survey provides the most comprehensive insight into clinician decision making concerning patients with CRPM being considered for CRS/IPC. The survey thoroughly gathered clinician responses regarding patient workup/assessment, selection for preoperative systemic therapy, preoperative and intraoperative selection for CRS/IPC, and consideration of prognosis and complications. Our data draws on expert opinion from 60 clinicians from 45 centres in 22 countries.

There are two other studies on this subject that warrant discussion. In 2018, Bushati et al.^8^ reported on a web-based survey of the Peritoneal Surface Oncology Group International (PSOGI) in 19 countries, which overlaps with our questioning related to preoperative staging and indications/contraindications for CRS/IPC. However, the authors placed much of their attention on the technique and method of CRS/IPC, while the focus of our questionnaire was that of patient selection for CRS/IPC. In 2020, Steffen et al.^10^ reported on decision-making algorithms regarding the use of hyperthermic intraperitoneal chemotherapy (HIPEC) after CRS as extrapolated from answers provided by twelve PSOGI executive committee experts using the objective consensus method. Again, this revealed strong variations among centres and was focussed on selection of patients to receive IPC. In contrast, our present study focusses heavily on the factors that influence clinical decision making and provide an analysis of patient- and disease-related aspects that may influence the delivery of this complex care.

Upon assessment of survey responses, several interesting trends were noted in each section of the survey. Regarding *preoperative workup/assessment and selection for preoperative systemic therapy*, good consensus existed regarding: (1) the use of a multidisciplinary team meeting for patient selection, (2) the use of computer tomography of chest, abdomen and pelvis for initial staging, and (3) the necessity of performing restaging following pre-surgery systemic chemotherapy administration. However, much variability existed regarding: (1) the use of magnetic resonance imaging (MRI), positron emission tomography (PET) scan and laparoscopy for staging; (2) the use of preoperative tumour marker measurements; and (3) the indications for systemic chemotherapy prior to consideration for surgery. Considering these trends in the context of currently available literature, good evidence is emerging for a combined modality approach to staging due to lower sensitivity of CT for < 5 mm peritoneal lesions (from 83 to 43%),^17^ while MRI (particularly diffusion weighted) improves sensitivity and specificity for peritoneal lesions and also allows for differentiation of ascites from solid tumour deposits as well as superior characterisation of liver lesions.^18^ In addition, exploratory laparoscopy can be used to evaluate occult carcinomatosis and reduce the incidence of unnecessary laparotomy^19^. Regarding systemic chemotherapy prior to CRS/IPC, recent systematic reviews have failed to show a survival benefit; however, some clinicians administer neoadjuvant chemotherapy and observe tumour response as a surrogate for the aggressiveness of peritoneal disease, thus guiding decisions about whether to proceed with CRS/IPC.^20,21^

Regarding *preoperative and intraoperative selection for CRS/IPC*, further interesting trends were identified. It was noted that molecular marker status (MSI/BRAF/KRAS) was regarded as less important than other factors, although many clinicians felt that this should be included in future prognostic scores to help aid decision making. This issue has already been addressed, to a certain degree, in a recent retrospective study of 524 patients from 6 tertiary centres by Schneider et al.,^12^ where KRAS and BRAF mutations were found to significantly impair survival, subsequently prompting creation of the BIOSCOPE risk score; although further prospective external validation of this score will be required to determine its impact on, and utility in, clinical decision-making algorithms. In addition, signet-cell histology of the tumour was considered by respondents to be “not” or only a “relative” contraindication for CRS/IPC, which is somewhat surprising given that signet-ring cell histology is associated with a particularly poor prognosis, with a median survival of < 3 months when treated with palliative care and also a negative prognostic impact when treated with CRS/IPC.^22,23^ Furthermore, while marked variations in reported PCI cut-offs were noted, some experts reported being content with conducting CRS/IPC in patients who had a PCI > 20, and a third of respondents did not alter PCI cut-offs in the presence of distant metastases. A direct relationship has been demonstrated between increased PCI and poorer overall survival, guiding several units to reserve CRS/IPC for those patients with a lower PCI.^24^ Furthermore, regarding treatment of those with CRPM and simultaneous liver metastasis, Lo Dico et al.^25^ showed that CRS/IPC with liver resection is feasible with acceptable morbidity and reasonably low postoperative mortality and significant survival benefit in those with limited peritoneal involvement only (PCI < 12). These findings, amongst others, indicate clear variations in clinical practice patterns.

Regarding *consideration of prognosis and complications following CRS/IPC*, a degree of variability regarding perceptions of acceptable rates of morbidity and mortality were identified, although there seemed to be some consensus for minimal acceptable overall survival being ≥ 24 months (71.7% of respondents), but less so for duration of recurrence-free survival. Furthermore, regarding acceptable rates of complication, two thirds of the cohort believed that post procedure mortality should remain ≤ 5%. A recent systematic review of 26 studies by Parikh et al. showed the mean weighted median disease-free survival was 15 months (range: 9–36 months) and median overall survival was 33.6 months (range: 12–63 months) among 20 studies. The mean weighted overall morbidity was 29% in 18 studies and mortality was 4% in 15 studies.^26^ Further to this, the recent multicentre randomised PRODIGE 7 trial by Quenet et al.^27^ reported a 30-day mortality rate of 2% and median overall survival of > 40 months with complete CRS regardless of the use of IPC. This study also reported occurrence of grade 3 or 4 adverse events in 37% of participants. In addition, we found that only 20% of respondents used the currently available prognostic scores in clinical practice, and a majority of these preferred PSDSS. Although several respondents reported reasonable justification for omitting use of prognostic scores in their practice, a good proportion reported that they would like to see prognostic scores/decision support tools used to aid decision making for several aspects of care in patients with CRPM, thus identifying a key area for future research.

Lastly, a subgroup analysis of the responses from those who practice in the context of a high-volume centre (≥ 50 cases per year) was performed. Although this yielded some interesting trends, it also emphasised that this subgroup is not immune to the dramatic variability in decision making seen throughout the general cohort. Some interesting trends included the higher percentage of respondents who have been consultant clinicians for only 5–10 years, possibly a result of evolving sub-specialised training and practice and centralisation of care for complex oncology. It was also noted that these respondents utilise MRI modalities more and are more selective with diagnostic laparoscopy, which may indicate a trend to more rapid uptake of technology when compared with the general cohort. In addition, this cohort did not consider any factor a contraindication for CRS/IPC, had higher PCI cut-offs and placed less importance on factors that traditionally make CRS more complex/difficult (extensive small bowel involvement, higher PCI, liver metastases), while conversely placing increased emphasis on those factors which act as a surrogate for tumour biology and aggressiveness (e.g. molecular markers, tumour markers, signet-cell pathology). This may indicate a trend towards more aggressive CRS and increased capability of managing more complex cases, while displaying an evolving understanding of tumour biological behaviour. In addition, the high-volume cohort tended to accept shorter recurrent-free survival times while demanding longer overall survival, which may indicate willingness to administer further lines of chemotherapy or even embark on reiterative CRS, if required, to extend survival.

The current survey explores several aspects of care of patients with CRPM while highlighting the many areas of marked variability in preoperative, intraoperative and postoperative decision making. It emphasises the need for development of standardisation and guidelines to facilitate a more homogeneous delivery of care in what is already a heterogeneous and challenging patient population. It highlights the trend towards centralisation of this complex oncological surgery to high-volume centres with evolving adoption of technology, advanced surgical techniques and multidisciplinary care. In addition, it displays the desire of clinicians for further evolution and development of decision-making tools to assist with prediction of outcomes to help guide patient management.

## Conclusion

CRS/IPC is performed for management of CRPM worldwide and on a relatively large scale. However, several uncertainties remain concerning almost every aspect of this treatment modality, resulting in marked variability in patient management and thus, potentially, outcomes. Given that no universally accepted guidelines exist, this international survey provides the most comprehensive insight into clinician decision-making trends regarding patient assessment, selection and management. This allows areas of variability to be more clearly defined and could potentially prompt development of initiatives to achieve consensus and standardisation of care in the future.

## Supplementary Information

Below is the link to the electronic supplementary material.

Supplementary file1 (DOCX 25 kb)

Supplementary file2 (DOCX 4199 kb)
