# Supplementary material for: Use of Prognostic Factors and Scores in Selection of Patients with Colorectal Cancer Peritoneal Metastasis (CRPM) for Cytoreductive Surgery and Intraperitoneal Chemotherapy (CRS/IPC): Results of an International Survey Among Oncologic Clinicians
Source: Ann Surg Oncol. 2023 Apr 5;30(6):3333–45. doi: 10.1245/s10434-022-12794-5 (PMC10175441; doi:10.1245/s10434-022-12794-5)
Supplement: Supplementary file 1 — Supplementary file1 (DOCX 25 kb) [file 10434_2022_12794_MOESM1_ESM.docx]

**Use of Prognostic Factors and Scores in Selection of Patients with Colorectal Cancer Peritoneal Metastasis (CRPM) for Cytoreductive Surgery and Intraperitoneal Chemotherapy (CRS/ IPC)**

**GENERAL INFORMATION**

Q1. In what country is your unit located?

| Country list |
| --- |

Q2. At which Hospital are you involved with CRS/ IPC for patients with CRPM? (This information will be kept confidential and is simply used to assess regional differences in practice within units located in the same country).

| Hospital name  State or province |
| --- |

Q3. What is your gender?

| Female  Male  Prefer not to disclose  Other (specify) |
| --- |

Q4. What is your area of specialty?

| Surgeon  Medical oncologist  Other (please specify) |
| --- |

Q5. Which of the following best describes your current level of training?

| Consultant  Fellow (Post Fellowship Training)  Trainee (Resident or Registrar)  Other (please specify) |
| --- |

Q6. If a consultant, how many years have you been in practice?

| < 5  5-10  >10 |
| --- |

Q7. How many years have you been involved in CRS/ IPC?

| < 5  5-10  >10 |
| --- |

Q8. How many CRS/ IPC operations does your unit perform each year?

| 0 - 9  10 - 19 | 20 - 49  ≥50 |
| --- | --- |

Q9. Does your unit have a pre-operative multidisciplinary team meeting (tumour board)?

| Yes  No |
| --- |

Q10. Which of the following best describes your pre-operative multidisciplinary (MDT) meeting?

| Dedicated CRS/IPC MDT  Colorectal (lower gastrointestinal) cancer MDT  Upper gastrointestinal cancer MDT  Gastrointestinal (upper and lower) cancer MDT  Other (please specify) |
| --- |

**PRE-OPERATIVE WORKUP**

Q11. Which of the following imaging modalities does your unit use for pre-operative staging of patients with CRPM?

| Computer Tomography (CT) Chest/ Abdomen/ Pelvis  Magnetic Resonance Imaging (MRI) Abdomen  Positron Emission Tomography (PET) Scan  Magnetic Resonance Imaging (MRI) Liver  None of the above  Other (please specify) |
| --- |

Q12. Do you use diagnostic laparoscopy for preoperative Peritoneal Cancer Index (PCI) calculation?

| Always  Never  Sometimes (please specify circumstances) |
| --- |

Q13. Do you routinely measure pre-operative tumour markers?

| Carcinoembryonic antigen (CEA)  Cancer antigen 19.9 (CA 19.9)  Cancer antigen 125 (CA 125)  None of the above  Other (please specify) |
| --- |

**NEOADJUVANT THERAPY**

Q14. Are your patients with CRPM considered for:(i) neoadjuvant/ preoperative systemic therapy, OR (ii) systemic therapy prior to consideration for surgery?

| Yes  No |
| --- |

Q15. Do your patients with metachronous/ synchronous CRPM tend to be given: (i) neoadjuvant/ preoperative systemic therapy, OR (ii) systemic therapy prior to consideration for surgery?

| Options: Always, Usually, Sometimes, Rarely, Never  Metachronous  Synchronous |
| --- |

Q16. In which patients with CRPM is it indicated to give:(i) neoadjuvant/ preoperative systemic therapy, OR (ii) systemic therapy prior to consideration for surgery?

| All patients  Those with unresectable disease  Those with high PCI. If so, what do you consider a high PCI? |
| --- |

Q17. In your unit, what is the preferred regimen for:(i) neoadjuvant/ preoperative systemic therapy, OR (ii) systemic therapy prior to consideration for surgery?

| Standard chemotherapy (FOLFOX, CAPOX/XELOX, FOLFIRI, FOLFOXIRI)  Targeted therapy (Cetuximab, Bevacizumab)  Pressurized intraperitoneal aerosol chemotherapy (PIPAC)  Unsure  Other (please specify) |
| --- |

Q18. Do you routinely restage patients post systemic therapy?

| Yes  No |
| --- |

Q19. Which modalities do you use to restage patients?

| Computer Tomography (CT) Chest/ Abdomen/ Pelvis  Magnetic Resonance Imaging (MRI) Abdomen  Positron Emission Tomography (PET) Scan  Magnetic Resonance Imaging (MRI) Liver  Other (please specify) |
| --- |

**FACTORS INFLUENCING PRE-OPERATIVE DECISIONS**

Q20. How important do you consider the following variables when deciding to offer CRS/IPC to patients with CRPM?

| Options: Not very important/ Not important/ Neutral/ Important/ Very important:  Patient age  Eastern Cooperative Oncology Group (ECOG) Performance Status​​  American Society of Anaesthesiologists (ASA) Physical Status  Histopathology - Signet cell histopathology  Histopathology - Poor tumour differentiation  Histopathology - Lymph node status of primary tumour  Short time from diagnosis/ resection of primary tumour to development of CRPM (<6 months)  Primary rectal cancer (vs colon cancer)  Peritoneal Cancer Index (PCI)  Presence of liver metastasis  Presence of lung metastasis  Retroperitoneal lymph node involvement  Liver hilar involvement  Gastric involvement  Extensive small bowel involvement  Ureteric involvement  Pelvic vascular involvement  Bony involvement  Pancreatic head involvement  Abdominal wall involvement  Inability to achieve a CC0 cytoreduction  Molecular marker - MSI  Molecular marker - KRAS  Molecular marker - BRAF  Raised tumour markers (CEA, CA19.9, CA125)  Other (please specify) |
| --- |

Q21. Which of the following do you consider to be contraindications for CRS/ IPC in patients with CRPM?

| Options: Not a contraindication/ Relative contraindication/ Absolute contraindication  Old age >80y.o.  Poor performance status - Eastern Cooperative Oncology Group (ECOG)  Poor Physical Status - American Society of Anaesthesiologists (ASA)  Histopathology - Signet cell histopathology  Histopathology - Poor tumour differentiation  Histopathology - Lymph node positive primary tumour  Short time from diagnosis/ resection of primary tumour to development of CRPM (<6 months)  Retroperitoneal lymph node involvement  Liver hilar involvement - Requiring reconstruction  Gastric involvement - Requiring gastric resection  Extensive small bowel involvement - Requiring extensive resection  Ureteric involvement - Requiring reconstruction  Pelvic vascular involvement - Requiring reconstruction  Bony involvement - Requiring resection +/- fixation  Pancreatic head involvement  Extensive abdominal wall involvement - Requiring major reconstruction  Inability to achieve a CC0 cytoreduction  Molecular marker - Microsatellite stable  Molecular marker - KRAS positive  Molecular marker - BRAF positive  Raised tumour markers (CEA, CA19.9, CA125)  Other (please specify) |
| --- |

Q22. Regarding Peritoneal Cancer Index (PCI), do you have a cut-off for offering CRS/ IPC to patients with CRPM in your unit?

| Yes  No |
| --- |

Q23. Is your PCI cut-off dependent on the presence of distant metastases (liver or lung)?

| Yes  No | |
| --- | --- |
| Q23. Is your PCI cut-off dependent on the presence of distant metastases (liver or lung)? | |
| Yes  No | |
| Q24. What is your PCI cut-off for offering CRS/IPC? | |
| ≤5  ≤10  ≤15  ≤20 | ≤25  ≤30  ≤35  ≤39 |
| Q25. In the absence of distant metastases, what is your PCI cut off for proceeding with CRS/IPC? | |
| ≤5  ≤10  ≤15  ≤20 | ≤25  ≤30  ≤35  ≤39 |

Q26. Please describe how the presence of distant metastases (liver or lung) effects your PCI cut-off for proceeding with CRS/IPC?

| Answer |
| --- |
| Q27. Regarding the presence of liver metastases, which of the following do you consider to be contraindications for CRS/ IPC in patients with CRPM? |
| Options: Not a contraindication/ Relative contraindication/ Absolute contraindication  Number of liver metastases ≤3  Number of liver metastases >3  Resectable requiring major liver resection  Non-resectable liver metastases  Other considerations (please specify) |
| Q28. Regarding the presence of pulmonary metastases, which of the following do you consider to be contraindications for CRS/ IPC in patients with CRPM? |
| Options: Not a contraindication/ Relative contraindication/ Absolute contraindication  Number of pulmonary metastases ≤3  Number of pulmonary metastases >3  Resectable requiring major lung resection  Non-resectable lung metastases  Other considerations (please specify) |

Q29. What other factors not yet outlined influence your pre-operative decision making?

| Answer |
| --- |

**FACTORS INFLUENCING INTRA-OPERATIVE DECISIONS**

| Q30. How important do you consider the following variables when deciding to proceed intra-operatively with CRS/IPC in patients with CRPM? |  |
| --- | --- |
| Options: Not very important/ Not important/ Neutral/ Important/ Very important:  Peritoneal Cancer Index (PCI)  Retroperitoneal lymph node involvement  Liver hilar involvement  Gastric involvement  Extensive small bowel involvement  Ureteric involvement  Pelvic vascular involvement  Bony involvement  Pancreatic head involvement  Abdominal wall involvement  Inability to achieve a CC0 cytoreduction  Other (please specify) |  |
| Q31. Which of the following do you consider to be contraindications for proceeding intra-operatively with CRS/ IPC in patients with CRPM? | |
| Options: Not a contraindication/ Relative contraindication/ Absolute contraindication  PCI which exceeds your previously stated cutt-offs  Retroperitoneal lymph node involvement  Liver hilar involvement - Requiring reconstruction  Gastric involvement - Requiring gastric resection  Extensive small bowel involvement - Requiring extensive resection  Ureteric involvement - Requiring reconstruction  Pelvic vascular involvement - Requiring reconstruction  Bony involvement - Requiring resection +/- fixation  Pancreatic head involvement  Extensive abdominal wall involvement - Requiring major reconstruction  Inability to achieve a CC0 cytoreduction  Other (please specify) | |

Q32. What other factors not outlined above influence your intra-operative decision making?

| Answer |
| --- |

**CONCLUDING QUESTIONS**

Q33. If you could predict the following for an individual patient, what would be the minimal acceptable time-period (in months) for you to feel that CRS/IPC is warranted?

| Recurrence free survival  Overall survival |
| --- |

Q34. Morbidity and mortality - At what percentage risk of the following would you recommend against CRS/HIPEC? (grade refers to Clavien Dindo classification)

| Grade 3 (requiring invasive intervention) or 4 (requiring ICU management) complications?  Grade 5 complications (peri-operative mortality)? |
| --- |

Q35. Are there other factors that would prompt you to recommend against CRS/IPC in patients with CRPM?

| Answer |
| --- |

Q36. Do you utilise any of the currently available prognostic scores (other than PCI alone) to decide in whom to recommend CRS/IPC?   Examples of prognostic scores include:

- Prognostic Score (PS)

- Peritoneal Surface Disease Severity Score (PSDSS)

- CPM Prognostic Surgical Score (COMPASS)

- COloREctal-Pc (COREP) or its modification (mCOREP)

- BIOlogical Score of COlorectal PEritoneal metastasis (BIOSCOPE)

| Yes  No |
| --- |

Q37. If so, which prognostic scores do you utilise?

| Prognostic Score (PS)  Peritoneal Surface Disease Severity Score (PSDSS)  Colorectal Peritoneal Metastases Prognostic Surgical Score (COMPASS)  COloREctal-Pc (COREP) or its modification (mCOREP)  BIOlogical Score of COlorectal PEritoneal metastasis (BIOSCOPE)  Other (please specify) |
| --- |
| Q38. If not, what are your reasons for not using these decision support tools? |
| Poor accuracy  Tedious to calculate  Inability to calculate preoperatively  Lack of clinical relevance  Lack of appropriate validation |
| Q39. What would you like to see a prognostic score used for? |
| Prediction/ prevention of open/ close laparotomy  Identification of patients who will achieve a survival benefit from CRS/ IPC  Prediction of response to chemotherapy  Avoidance surgery not likely to provide a survival benefit  Other (please specify) |

Q40. What are the relevant factors you think should be included in a prognostic score to help in decision making?

| Answer |
| --- |

Q41. Email address? (Used for the ensuring that survey is taken only once per participant. Will be kept confidential and not used for any correspondence).

| Answer |
| --- |
