# Supplementary material for: Use of Prognostic Factors and Scores in Selection of Patients with Colorectal Cancer Peritoneal Metastasis (CRPM) for Cytoreductive Surgery and Intraperitoneal Chemotherapy (CRS/IPC): Results of an International Survey Among Oncologic Clinicians
Source: Ann Surg Oncol. 2023 Apr 5;30(6):3333–45. doi: 10.1245/s10434-022-12794-5 (PMC10175441; doi:10.1245/s10434-022-12794-5)

**Adherence to Study Type Guidelines**

According to the Equator for Health Research Reporting Website, this manuscript and study type does not require a completed study guideline checklist.

As such, the Reporting guideline decision tree has been included below and the appropriate section circled in yellow to show this.


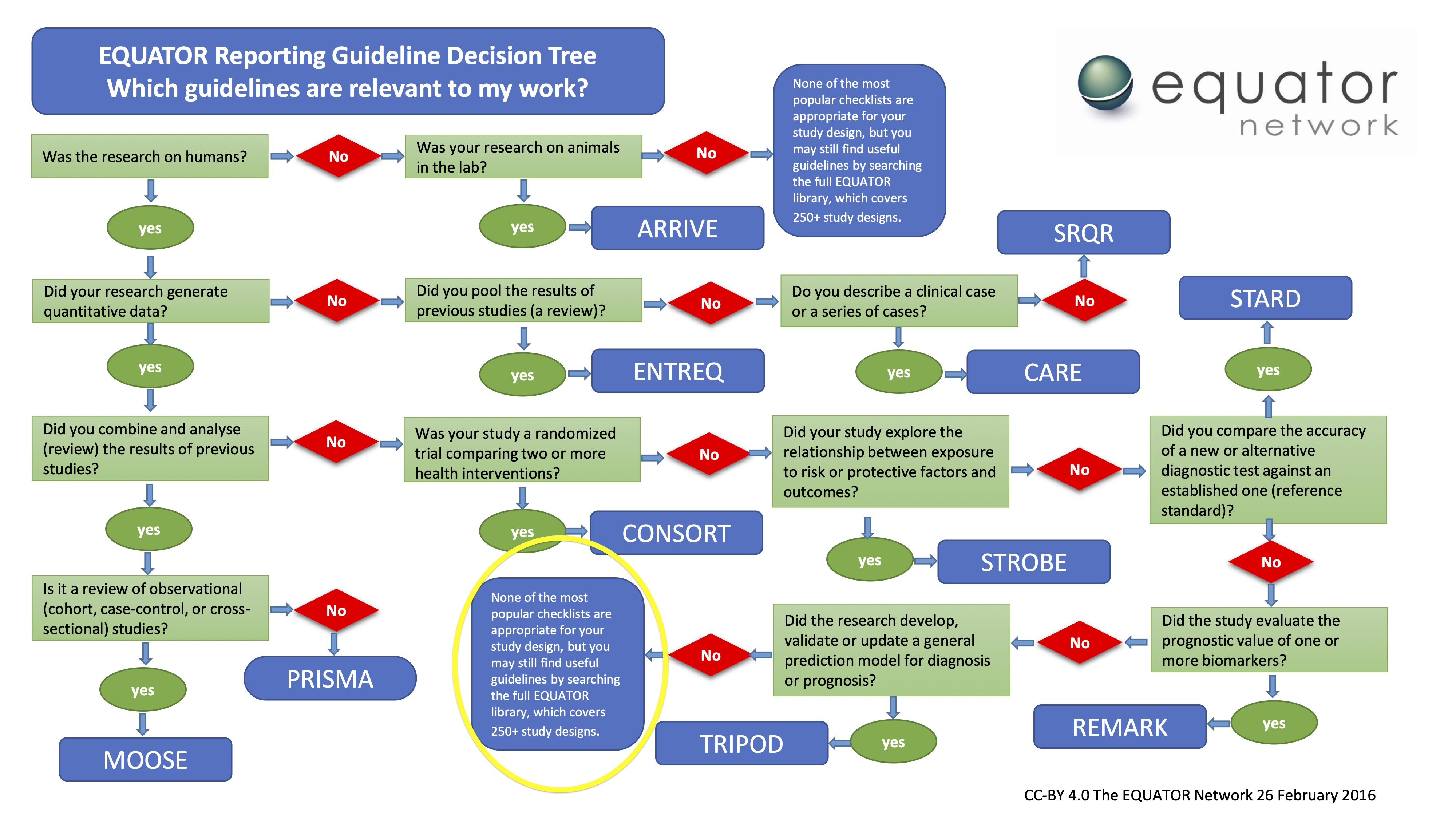

Supplement: Supplementary file 2 — Supplementary file2 (DOCX 4199 kb) [file 10434_2022_12794_MOESM2_ESM.docx]
